# Introduction of Cysteines in the Stalk Domain of Recombinant Influenza Virus N1 Neuraminidase Enhances Protein Stability and Immunogenicity in Mice

**DOI:** 10.3390/vaccines9040404

**Published:** 2021-04-19

**Authors:** Shirin Strohmeier, Juan Manuel Carreño, Ruhi Nichalle Brito, Florian Krammer

**Affiliations:** 1 Department of Microbiology, Icahn School of Medicine at Mount Sinai, New York, NY 10029, USA; shirin.strohmeier@mssm.edu (S.S.); jm.carreno@mssm.edu (J.M.C.); brito.n.ruhi@gmail.com (R.N.B.); 2 Department of Biotechnology, University of Natural Resources and Life Sciences, 1190 Vienna, Austria

**Keywords:** neuraminidase, N1, vaccine, stalk domain, cysteine mutant

## Abstract

Influenza virus surface glycoproteins represent the main targets of the immune system during infection and vaccination. Current influenza virus vaccines rely mostly on the hemagglutinin, requiring a close match between the vaccine and circulating strains. Recently, the neuraminidase (NA) has become an attractive target; however low immunogenicity and stability in vaccine preparations remain an obstacles. Here, we took advantage of the hypervariable stalk domain of the NA to introduce cysteines at different positions and to produce more stable multimeric forms of the protein. We generated 11 N1 constructs and characterized the proteins by performing sodium dodecyl sulfate polyacrylamide gel electrophoresis and by testing their enzymatic activity and representation of antigenic epitopes. Moreover, we evaluated their potential to induce a protective immune response in vivo and characterized the polyclonal antibody responses of immunized mice. We observed that the introduction of cysteines at certain positions led to the formation of stable N1 dimers, which are capable of inducing a strong antibody response characterized by neuraminidase inhibiting activity and protection of mice from high dose viral challenge. Overall, our results provide evidence for the feasibility of introducing stalk modifications to enhance the stability and immunogenicity of NA-based recombinant antigens.

## 1. Introduction

Influenza viruses belong to the *Orthomyxoviridae* family. They consist of enveloped particles carrying single-stranded negative sense RNA. The genome of the influenza A viruses consists of 8 segments that encode at least 11 structural and non-structural proteins [1,2]. These viruses carry two major glycoproteins on their surfaces, the hemagglutinin (HA) and the neuraminidase (NA). While the HA is immunodominant and undergoes constant antigenic drift, particularly in the head domain [3], the NA seems to be relatively less prone to antigenic drift over time [4]. The influenza virus NAs can be classified into 4 different groups and 11 subtypes: group 1 (N1, N4, N5, N8), group 2 (N2, N3, N6, N7, N9), influenza B virus NAs (B-NAs), bat-like NAs, and the Wuhan spiny eel virus NA [5,6,7,8]. These proteins are type 2 integral membrane proteins, which consist of four identical monomers forming a homotetramer [9]. A single monomer consists of four different domains: a cytoplasmic tail, a transmembrane domain, a variable stalk, and a globular head domain bearing the enzymatic active site [10]. Through its enzymatic activity, the NA cleaves sialic acid residues from newly formed virions budding from the host cell surface [11] and destroys decoy receptors in the mucosa, such as sialic acids on N-like glycans on mucins [12], hence making this protein a crucial component of the virus during the replication cycle. Indeed, the enzymatic activity of the NA represents one of the main targets of antiviral drugs to treat influenza virus infections [13]. At the molecular level, these drugs prevent virions from detaching from the infected host cell, resulting in their aggregation on the cell surface [14]. Consequently, these drugs are able to reduce viral shedding and improve influenza symptoms if administered at early stages of the infection [15].

The viral surface proteins represent the main targets of the immune system during infection and vaccination. Although the HA has been historically targeted because antibodies against it can neutralize incoming virus, recent studies point towards the NA as an attractive antigen for induction of protective immune responses [5,8,16,17]. Indeed, antibodies against the NA have been described as an independent correlate of protection [18,19,20,21]. However, obstacles need to be overcome to improve the relatively low immunogenicity and stability of the NA in vaccine preparations [22]. In addition to NA’s immuno-subdominant role with respect to the HA [8,23], vaccine formulations containing live attenuated or inactivated influenza viruses lack standardized NA amounts [24,25]. Furthermore, the NA might display an improperly folded conformation, hence current vaccines elicit suboptimal responses against this antigen [22]. The amount of NA on the viral surface is approximately 20% of the amount of HA [9]. Based on the subtype and strain of the NA, the length of the stalk domain also varies [26] and these differences impact the viral replication and overall virulence of different strains [27,28,29].

Even though the stalk domain of the NA is variable and can differ significantly even within one subtype, it contains conserved structural features, including a specific cysteine residue and a potential glycosylation site [10,26]. These cysteine residues within the stalk domain form disulfide bonds, which are crucial for the formation of a stable tetrameric protein [26,30] and are responsible for the formation of dimers between the monomers, making the NA “a dimer of dimers”. Given that the stalk domain of the NA is variable, and therefore is tolerant to mutations [27], specific residues can be modified to potentially enhance recombinant protein stability and favor the formation of multimers, without the use of exogenous tetramerization domains.

In nature, the process of NA tetramerization is mediated partially by the transmembrane domain, as well as by interactions of the protomers in the head domain [31], but mimicking this process in vitro during recombinant protein production still represents a challenge. Hence, to evaluate whether mutating discreet regions within the stalk domain can increase protein stability and tetramer formation, we generated 11 N1 mutant constructs, which introduce cysteines at different positions within the stalk. Specifically, we aimed at connecting two soluble NA dimers by introducing additional cysteines to form tetramers via additional disulfide bonds. We characterized the recombinant NA proteins at the structural and functional levels and evaluated their potential to induce protective immune responses in vivo.

## 2. Materials and Methods

### 2.1. Cells and Viruses

Sf9 (*Spodoptera frugiperda*) insect cells were grown in *Trichoplusia ni* medium–Fred Hink (TNM-FH, Gemini Bioproducts, Sacramento, CA, USA) insect cell medium supplemented with 1% penicillin–streptomycin antibiotics mix (100 U/mL of penicillin, 100 µg/mL streptomycin, Gibco, Gaithersburg, MD, USA), 0.1% pluronic F-68 (Sigma-Aldrich, St. Louis, MO, USA), and 10% fetal bovine serum (FBS, Gibco). For passaging of the baculoviruses in Sf9 cells, TNM-FH insect medium (1% penicillin/streptomycin, 1% pluronic F-68, 3% FBS) was used. BTI-*TN*-5B1-4 (*Trichoplusia ni*, High Five) cells were passaged in serum-free Express Five insect cell medium (Gibco) containing 1% penicillin–streptomycin and 1% L-glutamine (Gibco). The challenge virus A/Singapore/GP1908/2015 (H1N1) was grown in 10-day-old embryonated chicken eggs (Charles River laboratories, Wilmington, MA, USA). Viral titers were determined by performing a standard plaque assay as previously described [31]. To purify the virus for the neuraminidase inhibition (NI) assay, A/Michigan/45/2015 was grown in 10-day-old eggs for 2 days at 37 °C, harvested, and purified using a 30% sucrose gradient in 1× NTE buffer (0.5 mM NaCl, 10 mM Tris-HCl pH 7.5, 5 mM ethylenediaminetetraacetic acid (EDTA)).

### 2.2. Constructs

The recombinant N1 constructs (A/Michigan/45/2015 H1N1) were expressed using the baculovirus expression system [32]. We designed 11 different constructs: AA82-388, containing only the globular head domain of the NA and a 10 amino acid stalk overhang; AA46-388, consisting of the wild type N1 with the full-length stalk domain (62aa); AA46-388 (C49A), as a monomer control in which the cysteine at position 49—which is essential for formation of stable dimers—was mutated to alanine [31]; constructs AA46-388 (T48C), AA46-388 (N50C), AA46-388 (T48C, N50C), AA46-388 (A76C), AA46-388 (Q78C), AA46-388 (V81C), and AA46-388 (W61C), containing cysteine mutations at different locations in the stalk domain; AA46-388 (VASP), as a tetramer control containing a full-length stalk domain (62aa), as well as a vasodilator-stimulated phosphoprotein (VASP) tetramerization domain [33]. All constructs were cloned into a baculovirus shuttle vector containing an N-terminal signal peptide, followed by a hexahistidine purification tag and a thrombin cleavage site. The baculoviruses were passaged in Sf9 cells to obtain high viral titers and were used to infect High Five cells. The recombinant proteins were purified from cell culture supernatants 72 h post-infection and purified as previously described [34]. The protein concentration was measured using Quick Start™ Bradford 1× Dye Reagent (BioRad, Hercules, CA, USA) and protein solutions were stored at −80°C until further usage.

### 2.3. Sodium Dodecyl Sulfate Polyacrylamide Gel Electrophoresis (SDS-PAGE)

To determine protein integrity, 1.5 ug of the respective recombinant N1 mutant protein was mixed 1:1 with 2× Laemmli loading buffer (Bio-Rad). For reducing conditions, loading buffer was supplemented with 5% beta-mercaptoethanol. To assess the extent of protein multimerization, samples were treated with the crosslinker bis-sulfosuccinimidyl suberate (BS3, ThermoFisher, Waltham, MA, USA) according to the manufacturer’s instructions. Bovine serum albumin (BSA) was used as a monomeric control and recombinant A/Michigan/45/2015 N1 protein containing only the globular head domain and the VASP tetramerization domain was used as control for tetramerization. Samples were heated at 95°C for 15 min prior to loading them on a sodium dodecyl sulphate polyacrylamide gel (4–20% Mini-PROTEAN^®^ TGX™ Precast Protein Gels, BioRad). Gels were stained with Coomassie blue (ThermoFisher) for 1 h at RT and de-stained with distilled water to visualize the proteins.

### 2.4. Enzyme-Linked Immunosorbent Assay (ELISA)

Ultra-high binding polystyrene 96-well plates (Immulon 4 HBX plates, ThermoFisher) were coated with 50 µL/well of recombinant protein (2 µg/mL) or purified virus (6 µg/mL) in phosphate-buffered saline (PBS) (pH = 7.4, Gibco) overnight at 4°C. Plates were washed 3 times with PBS plus 0.1% Tween 20 (TPBS) using an automated plate washer (AquaMax 2000, Molecular Devices, San Jose, CA, USA), then blocked with 100 µL/well of TPBS containing 3% non-fat dry milk (AmericanBio, Canton, MA, USA) for 1 h at room temperature (RT). The blocking solution was removed and monoclonal antibodies (mAbs) diluted in 1%-milk TPBS were added to the plates (initial concentration of 30 µg/mL followed by 1:3 serial dilutions). To assess whether the recombinant N1 mutant proteins were correctly folded and properly displayed antigenic determinants, two previously described human monoclonal antibodies were used for binding assays: 1G01, a broad anti-NA antibody; and 1000-1D05, an N1-specific monoclonal antibody [35]. As a negative control, an anti-Lassa virus glycoprotein antibody, KL-AV-1A12, was used [36].

For serum ELISAs, samples were initially diluted (1:50) in 1%-milk TPBS and further serially diluted (1:3). The serum from mice vaccinated with an irrelevant protein was used as negative control and the anti-NA mouse monoclonal antibody 4A5 was used as positive control. Plates containing the mAbs or sera were incubated for 2 h at RT, followed by three washes with 100 µL/well of TPBS. The respective secondary antibody consisting of anti-mouse IgG heavy and light chain peroxidase-conjugated (Rockland, Limerick, PA, USA) or anti-human IgG Fab-specific horseradish-peroxidase (HRP) conjugated (Sigma-Aldrich) antibody was diluted 1:3000 in 1% milk/TBPS and added to the plates (100 µL/mL) for 1 h at RT. Plates were washed three times with TBPS (100 µL/well) and incubated at RT with 100 µL/well of SigmaFast *o*-phenylenediamine dihydrochloride (OPD) developing solution (Sigma-Aldrich). The reaction was stopped by adding 50 µL/well of 3 M hydrochloric acid (HCl). The plates were read using a Synergy H1 hybrid multimode microplate reader (BioTek, Winooski, VT, USA) at an optical density of 490 nm. The data were analyzed using GraphPad Prism 7 software and values were expressed as the area under the curve (AUC).

### 2.5. NA Star Assay

The NA enzymatic activity was determined by using the NA-Star™ Influenza Neuraminidase Inhibitor Resistance Detection Kit (ThermoFisher). Briefly, the recombinant N1 mutant proteins were diluted to a starting concentration of 10 µg/mL then serially diluted (1:3). The assay was performed according to the manufacturer’s instructions. As a positive control, recombinant A/Michigan/45/2015 N1 containing the VASP tetramerization domain was used.

### 2.6. Neuraminidase Inhibition (NI) Assay

The NI assay was performed as previously described [22]. Briefly, 96-well, flat bottom, non-sterile Immulon 4 HBX plates (ThermoFisher) were coated with 150 µL of 50 µg/mL fetuin (Sigma-Aldrich) at 4 °C overnight. Serum samples were diluted in PBS (1:50) and further serially diluted (1:3). A/Michigan/45/2015 H1N1 virus was diluted in PBS and added to the serum dilution plates at twice the 50% effective concentration (EC_50_) to every well. Serum–virus plates were incubated at RT for 1 h 45 min while shaking. During this time, the fetuin-coated plates were blocked for 1 h at RT with 5% BSA in PBS and washed 3 times with TPBS. The serum–virus mix was added to the fetuin-coated plates (100 µL/well) and plates were incubated for 2 h at 37 °C. After 3 washes with TPBS, plates were incubated for 1 h 45 min with 100 µL/well of 5 µg/mL peanut agglutinin (PNA) conjugated to HRP (Sigma-Aldrich). Plates were washed 3 times with TPBS, then SigmaFast OPD developing solution was added to the wells (100 µL/well). After 7 min of incubation, the reaction was stopped by adding 50 µL/well of 3 M HCl. The optical density (OD) was measured at 490 nm using a Synergy 4 plate reader (BioTek). The data were analyzed by using GraphPad Prism 7 software and values were expressed as percentages of inhibition.

### 2.7. Animal Study

Blood samples were obtained by sub-mandibular bleeding. Female 6–8 week old BALB/c mice (Jackson Laboratories, Bar Harbor, ME, USA) were administered intramuscularly (I.M., *n* = 5 per group) with 3 µg of recombinant NA protein mixed 1:1 with AddaVax (InvivoGen, San Diego, CA, USA). The negative control group received 3 µg of irrelevant B/Malaysia/2506/04 HA protein via the same route. Three weeks post-prime, the mice were bled and boosted with the same antigen dose. Four weeks post-boost, the mice were bled and challenged with 25 times the 50% lethal dose (LD_50_) of A/Singapore/GP1908/2015 H1N1 virus diluted in PBS. For the second experiment, mice were vaccinated following the same regimen but only using the AA46-388 (T48C + N50C) and AA46-388 constructs. Subsequently, mice were challenged with 100 times the LD_50_ of A/Singapore/GP1908/2015 H1N1 virus. Survival and weight loss were monitored in both experiments over 14 days. Mice were euthanized if they lost 25% of their respective initial weight.

## 3. Results

### 3.1. Generation and Characterization of Recombinant Neuraminidases with Stalk-Specific Mutations

**Figure 1:**
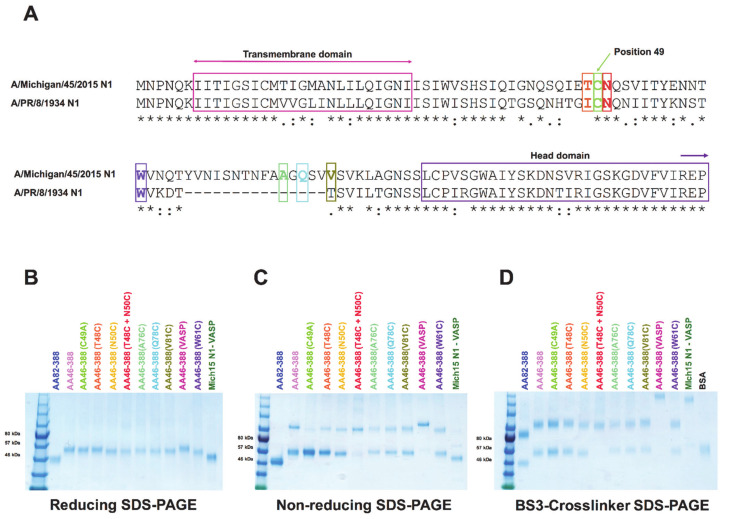
Sequence and structural analyses of recombinant NA constructs. A sequence alignment between the stalk domain of A/Michigan/45/2015 N1 and A/PR/8/1934 N1 is shown (* indicates identical amino acids, : indicates similar amino acids, . indicated by the dotted line). The recent A/Michigan/45/2015 strain contains a 15aa longer NA stalk domain compared to the older A/PR/8/34 isolate. The transmembrane domain and the start of the globular head domain are highlighted with arrows. Sites chosen for the introduction of an additional cysteine mutation are shown in different colors (**A**). NA constructs were analyzed by reducing SDS-PAGE under denaturing conditions to identify monomers (**B**); by non-reducing SDS-PAGE to show dimers (**C**); or treated with a BS3 crosslinker (**D**) to evaluate the formation of tetramers.

A correctly folded neuraminidase (NA) structure is needed to induce protective antibody responses [36]. However, current vaccine formulations do not optimally display a natural protein conformation, as opposed to NA present in circulating viruses [22,24]. Despite attempts to generate an NA-based recombinant vaccine, these efforts have been hampered by difficulties in establishing a proper quaternary conformation. Here, we designed several NA constructs consisting of the globular NA head domain only (AA82-388); a wild type N1 with the full-length stalk domain (AA46-388)); a monomer control in which the cysteine at position 49—which is essential for formation of stable dimers—was mutated to alanine [31] (AA46-388 (C49A)); several constructs containing cysteine mutations in different locations of the stalk domain (AA46-388 (T48C), AA46-388 (N50C), AA46-388 (T48C + N50C), AA46-388 (A76C), AA46-388 (Q78C), AA46-388 (V81C), AA46-388 (W81C)) (Figure 1A); and a tetramer control containing a full-length stalk domain along with the VASP tetramerization domain (AA46-388 (VASP)) (Table 1).

We analyzed the integrity and conformation of all of the constructs by running SDS-PAGE gels under reducing and non-reducing conditions and after crosslinking with BS3 (Figure 1B–D). Under reducing conditions, the recombinant NAs were detected at their monomeric conformation with an approximate molecular weight of 55 kDa (Figure 1B). Since AA82-388 and the control Mich15 N1-VASP only contained the globular head domain, the molecular weight was around 45 kDa. AA46-388 (VASP), which includes a slightly longer stalk domain, displays a molecular weight of 57 kDa (Figure 1B). Under non-reducing conditions, most of the constructs displayed a dimeric conformation of approximately 110 kDa (Figure 1C). Finally, the use of BS3 crosslinker, which crosslinks primary amines, resulted in the visualization of NA tetramers of approximately 220 kDa for the constructs carrying the VASP domain (Figure 1D).

**Figure 2:**
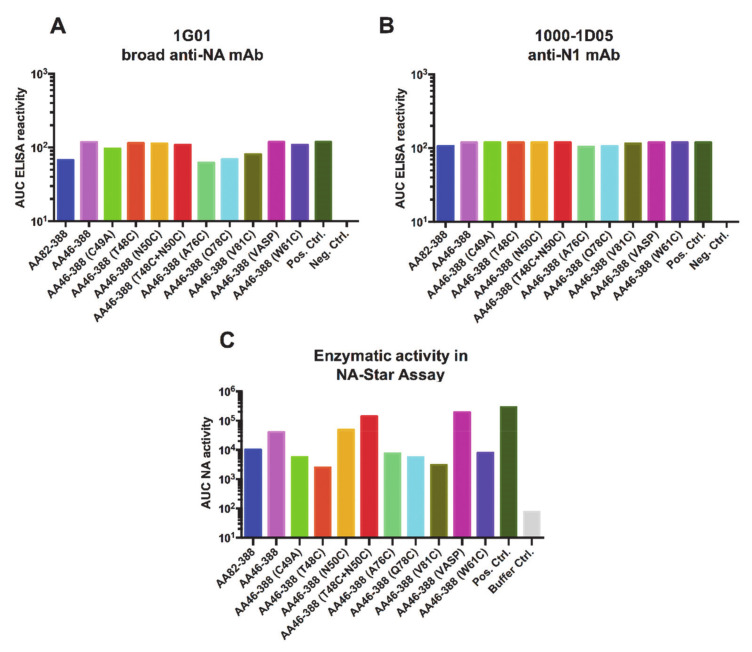
Antigenicity and enzymatic activity of recombinant NA constructs. ELISA plates were coated with recombinant NA proteins carrying the different stalk mutations. Binding of the broadly reactive NA monoclonal antibodies 1G01 (**A**) and 1000-1D05 (**B**) was tested. The enzymatic activity of the NA constructs was assessed using the NA-Star assay. (**C**). The tetrameric Mich15 N1 containing the VASP domain was used as a positive control (in all three panels), while mAb KL-AV-1A12 (anti-arenavirus glycoprotein mAb) was used as a negative control in (**A**,**B**).

To further characterize the conformation and functionality of the recombinant NAs, we tested binding of two anti-NA monoclonal antibodies, which had been previously reported: 1G01, a broad anti-NA mAb; and 1000-1D05, an anti-N1 specific mAb (35). No significant differences were observed in the mAb binding profiles for the different constructs (Figure 2A,B). To assess the enzymatic activity of the constructs, we performed an NA-Star assay. As a positive control for both assays, the tetrameric Mich15 N1 containing the VASP domain was used. The anti-Lassa virus glycoprotein antibody 1A12 (36) was used as a negative control. Constructs AA46-388 (T48C), AA46-388 (A76C), AA46-388 (Q78C), AA46-388 (V81C), and AA46-388 (W61C), which contain single cysteine mutations, showed the lowest enzymatic activities, probably based on unfavorable conformation of the respective constructs. The monomeric variant A46-388 (C49A) and the head only construct AA82-388 showed low enzymatic activity, as expected. Interestingly, once AA46-388 (T48C) was combined with AA46-388 (N50C), the enzymatic activity increased significantly, making the construct (AA46-388 (T48C + N50C) the one with the highest enzymatic activity except for the controls. These results indicate that the recombinant NAs not only exhibit a multimeric conformation, but that they also retain enzymatic activity.

### 3.2. In Vivo Protection Induced by Vaccination with Recombinant Constructs in the Mouse Model

**Figure 3:**
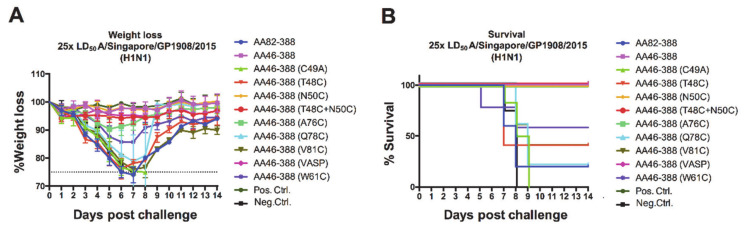
Weight loss kinetics and survival monitoring of vaccinated mice following H1N1 challenge. Mice were vaccinated with each of the NA constructs (*n* = 5 per group) in a prime-boost regimen. Here, 3 ug of the respective recombinant protein adjuvanted with AddaVax was administered. As negative and positive controls, the Lassa virus glycoprotein and the tetrameric Mich15-VASP protein were administered. Mice were then challenged with 25× LD_50_ of A/Singapore/GP1908/2015 virus. Weight loss was monitored over 14 days post-challenge (**A**). Mice were euthanized when they lost 25% of their initial weight. Survival curves are shown (**B**).

In addition to the immuno-subdominant role of the NA [8,23], vaccine formulations containing live attenuated or inactivated influenza viruses lack standardized amounts of NA [24,37] and might display improperly folded NA. Hence, these types of vaccines elicit limited responses against the NA [22]. To test whether NA constructs carrying different mutations in the stalk elicited protective immune responses *in vivo*, we administered mice with the recombinant NAs generated in a prime boost vaccination regimen and challenged them with 25 times the LD_50_ of A/Singapore/GP1908/2015 (H1N1) virus. We observed different levels of protection after vaccination; mice receiving AA46-388, AA46-388 (N50C), AA46-388 (T48C + N50C), and AA46-388 (A76C) did not display significant weight loss, while all other mice experienced significant weight drops at days 6–7 post-challenge (Figure 3A). Importantly, unvaccinated mice (negative control) and mice receiving the monomer control (AA46-388 (C49A)) or the monomeric NA head only (AA82-388) did not survive the challenge and succumbed to infection around days 8–9 (Figure 3B). Overall, weight loss and survival data evidenced the need for a stable multimeric NA to induce protection against lethal challenge.

### 3.3. Characterization of Sera from Mice Vaccinated with NA Constructs

**Figure 4:**
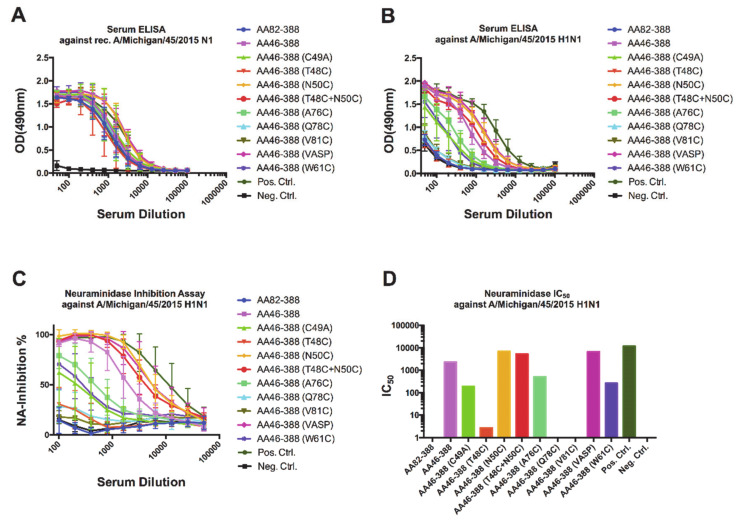
Characterization of anti-NA antibody responses in sera from vaccinated samples. Pre-challenge samples (after the 2nd vaccination) were obtained from the different groups of mice. Antibodies in serum able to bind to a recombinant tetrameric Mich15-VASP protein (**A**) or to the A/Michigan/45/2015 H1N1 virus (**B**) were measured by ELISA. The ability of serum antibodies to inhibit the neuraminidase enzymatic activity was assessed using the A/Michigan/45/2015 virus (**C**). The 50% inhibitory concentration (IC_50_) for each pool of serum samples is shown (**D**).

To understand the mechanisms by which antibodies confer protection *in vivo*, we collected pre-challenge serum samples from vaccinated mice and assessed binding to a recombinant A/Michigan/45/2015 N1 protein (Figure 4A) or purified A/Michigan/45/2015 H1N1 virus (Figure 4B). Interestingly, binding to the recombinant A/Michigan/45/2015 N1 VASP protein did not show substantial differences among the different groups. In contrast, binding to purified virus showed marked differences among the constructs, with high detectable levels of antibodies induced by AA46-388, AA46-388 (N50C), AA46-388 (T48C + N50C), and AA46-388 (VASP); and intermediate levels induced by AA46-388 (A76C) and AA46-388 (W81C). The antibody binding profile to purified virus was mirrored by the NI activity of the antibodies contained in the sera (Figure 4C), with the highest NA IC_50_ values for AA46-388, AA46-388 (N50C), AA46-388 (T48C + N50C), and AA46-388 (VASP); and intermediate values for AA46-388 (A76C) and AA46-388 (W81C) (Figure 4D). Importantly, the NA constructs with the highest IC_50_ values had NI levels similar to the tetrameric control proteins AA46-388 (VASP) and Mich15 N1 containing the VASP domain.

**Figure 5:**
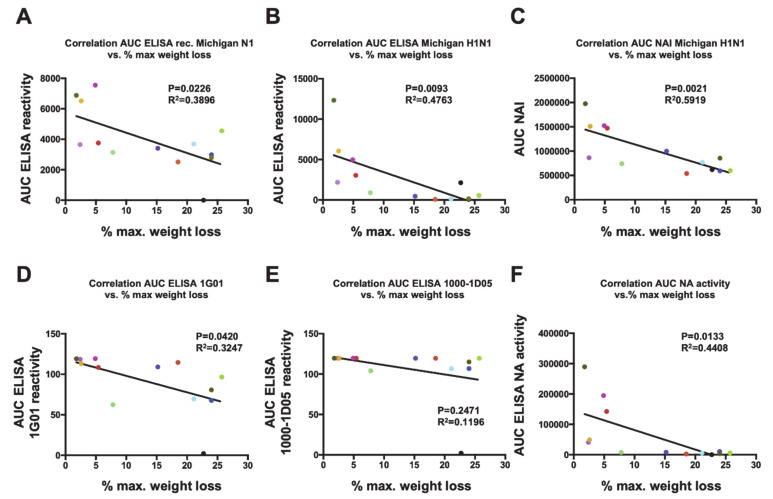
Correlations between weight loss and antibody responses. Correlation analyses of the maximum percentage of weight loss (MPWL) after vaccination with the different NA constructs and viral challenge (refer to Figure 3) were performed against different parameters. MPWL vs. area under the curve (AUC) obtained from an ELISA using the recombinant tetrameric Mich15-VASP protein (**A**) or purified A/Michigan/45/2015 virus (**B**). MPWL vs. the AUC obtained from an NI assay using purified A/Michigan/45/2015 (**C**). MPWL vs. ELISA binding of the broadly reactive NA monoclonal antibodies 1G01 (**D**) and 1000-1D05 (**E**) to the respective recombinant NAs. MPWL vs. the enzymatic activity of the respective NAs (**F**). The colors of the dots are consistent with groups in the preceding figures.

Importantly, the levels of antibodies measured by ELISA and inhibition of the NA enzymatic activity correlated well with reduction of disease severity *in vivo*, as measured by the maximum percentage of weight loss (MPWL). A negative correlation between the MPWL vs. antibody levels against purified A/Michigan/45/2015 H1N1 virus (r2 = 0.4763, *p* = 0.0093; Figure 5B) or vs. the NI activity (NAI; r2 = 0.5919, *p* = 0.0021; Figure 5C) was observed. Although a negative correlation between the MPWL and antibody levels against recombinant A/Michigan/45/2015 N1 (r2 = 0.3896, *p* = 0.0226; Figure 5A) was detected, this was less robust. Moreover, reduction in MPWL did not correlate well with binding of the broadly reactive NA monoclonal antibodies 1G01 (D) and 1000-1D05 (E) to the respective recombinant NAs in ELISA. However, a negative correlation of MPWL with the enzymatic activity of the respective NAs was found and was robust (Figure 5F).

**Figure 6:**
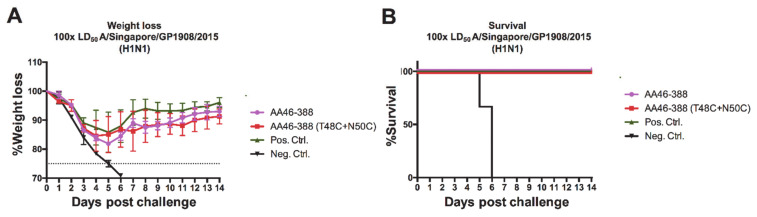
Challenge of vaccinated mice with a highly lethal dose of H1N1 virus. Mice were vaccinated with each of the indicated NA constructs (*n* = 5 per group) in a prime boost regimen. Here, 3 ug of the respective recombinant protein adjuvanted with AddaVax was administered. As negative and positive controls, the Lassa virus glycoprotein and the tetrameric Mich15-VASP protein were administered. Mice were then challenged with 100× LD_50_ of A/Singapore/GP1908/2015. Weight loss was monitored over 14 days post-challenge (**A**). Mice were euthanized when they lost 25% of their initial weight. Survival curves are shown (**B**).

Finally, to assess the strength of the protective effect induced by vaccination with the recombinant NAs, we administered to mice one of the constructs that displayed one of the best protective profiles—the double cysteine mutant AA46-388 (T48C + N50C)—and challenged them with 100 times the LD_50_ of A/Singapore/GP1908/2015 (H1N1). Despite the transient weight loss that peaked at 5 days post-infection (Figure 6A), mice were protected against a highly lethal viral dose (Figure 6B). Overall, these results indicate that vaccination with the recombinant NA constructs designed in this study, which carry specific cysteine mutations in the stalk domain, enhances the stability and immunogenicity of the NA *in vivo*, without the need for an exogenous tetramerization domain.

## 4. Discussion

The neuraminidase is a “dimer of dimers”. The first dimer is held together by a disulfide bond in the stalk domain of the protein. Dimerization of the two dimers to a tetramer is likely facilitated by both the transmembrane domains and by interactions of the protomers in the head domain [31]. The enzymatic activity of the NA is optimal in the tetramer form, while it is reduced in the dimer state and very low in the monomeric state. Importantly, multimerization also has an important role in antigenicity and protection by NA constructs as vaccines. We have previously shown—and confirmed here—that N1 constructs that are monomeric are not protective against challenge, even though they induce antibody responses [38]. In contrast, tetrameric NAs induce strong, protective antibody responses. The reason for this could be that the monomer displays epitopes that are usually hidden within the interface of the protomers and which may be immune-dominant but non-protective and not accessible in wild-type NAs. Interestingly, the strongly protective mAbs 1G01 and 1000-1D05 had no difficulties in binding to all constructs, suggesting that protective epitopes are present on monomers but might not be targeted appropriately by B-cells when these antigens are used for immunization.

Here, we aimed to connect two soluble NA dimers by introduction of different disulfide bonds to form a tetramer. While some weak tetramer formation was observed for construct AA46-388 (T48C + N50C), we failed to produce stable tetramers by introduction of an additional disulfide bond in the stalk domain. However, we showed that N1 dimers are capable of inducing strong antibody responses that show NI activity and protect mice, even from a very high dose challenge. Dimers are still outperformed by the tetramer in terms of protective immune responses; however, even responses induced by dimers may contribute to protection from disease. Another interesting observation made here is that introduction of an additional cysteine towards the head domain of the NA is counterproductive. This is likely due to interference of this free cysteine with disulfide bonds that are usually formed within the head domain of the monomer.

Lessons learned here will help us to better design novel soluble recombinant NA vaccine candidates without the need for exogenous tetramerization elements such as the VASP domain. Data regarding cysteine positions in the current constructs and their impact on dimerization and tetramerization will inform next-generation cysteine-mutant-based constructs, which are already in development. Ultimately, we aim to test such constructs as trivalent (N1, N2, B-NA) standalone vaccines or as supplement to current inactivated influenza virus vaccines in humans. Importantly, while the tetramerization mechanism is conserved across NA subtypes, additional construct designs will need to be explored for N2 and B-NA antigens.

## Figures and Tables

**Table 1:** Description of N1 mutant constructs.

Construct Name	Description of Mutation
AA82-388	Neuraminidase head only
AA46-388	Neuraminidase head + 36 aa of stalk domain
AA46-388 (C49A)	Monomeric control
AA46-388 (T48C)	Cysteine mutation in the stalk at position 48
AA46-388 (N50C)	Cysteine mutation in the stalk at position 50
AA46-388 (T48C + N50C)	Cysteine double-mutation in the stalk at position 48 and 50
AA46-388 (A76C)	Cysteine mutation in the stalk at position 76
AA46-388 (Q78C)	Cysteine mutation in the stalk at position 78
AA46-388 (V81C)	Cysteine mutation in the stalk at position 81
AA46-388 (VASP)	Neuraminidase head + 36 aa of stalk domain + VASP domain
AA46-388 (W61C)	Cysteine mutation in the stalk at position 61

## Data Availability

The data presented in this study are available on request from the corresponding author.
